# Transformative experiential learning in psychomotor skill development: a model informed by international nursing students’ learning experiences

**DOI:** 10.3389/fmed.2026.1796125

**Published:** 2026-05-22

**Authors:** Esra Sezer, Sevim Şen Olgay, Hilal Yıldız Çelik, Esra Uğur, Ükke Karabacak

**Affiliations:** Department of Nursing, Acibadem Mehmet Ali Aydinlar University, Istanbul, Türkiye

**Keywords:** experiential learning, international nursing students, psychomotor skills, qualitative study, simulation-based education, transformative learning

## Abstract

**Aim:**

This study aimed to examine how international undergraduate nursing students experience psychomotor skill learning within culturally diverse simulation-based education and to elucidate the cognitive, emotional, and developmental processes shaping this learning. Drawing on Mezirow’s Transformative Learning Theory and Kolb’s Experiential Learning Model, the study explored how early laboratory-based training contributes to professional competence relevant to safe and effective healthcare practice.

**Methods:**

An interpretive qualitative design was employed. Semi-structured interviews were conducted with 20 international undergraduate nursing students enrolled in a Turkish-medium nursing program. Data were analyzed using a hybrid thematic approach that integrated inductive thematic analysis with theory-informed deductive mapping to capture both lived experiences and underlying learning mechanisms.

**Results:**

Four main themes and 14 subthemes were identified, reflecting a progressive learning process. Integrating these findings led to the development of the Transformative Experiential Learning Model, which conceptualizes a developmental trajectory from initial disorientation to conceptual understanding and professional confidence. Early learning was characterized by linguistic barriers, cultural differences, unfamiliar technological platforms, performance anxiety, and challenges with medical terminology. Visual scaffolding, structured instructional guidance, immediate feedback, and peer support facilitated learners’ reinterpretation of challenges through repeated practice, observation, and reflection.

**Conclusion:**

The findings indicate that transformative learning related to professional identity formation and clinical reasoning can begin within simulation-based education prior to clinical placement, positioning the simulation laboratory as a critical early site for healthcare workforce preparation. Culturally responsive instruction, multimodal educational supports, and psychologically safe learning environments are essential for fostering international nursing students’ psychomotor skill development. Strengthening these elements may enhance readiness for clinical practice, reduce transition-related performance anxiety, and support safer and more effective healthcare delivery. It should be noted that these findings reflect early transformative processes and the development of an emerging professional identity within a preclinical context, rather than a full epistemic transformation in the Mezirow sense.

## 1 Introduction

Global trends in higher education show a significant increase in international student mobility. According to the Organisation for Economic Co-operation and Development (OECD), the number of international students enrolled in OECD countries rose from 3 million in 2014 to 4.6 million in 2022, a growth that persisted even during the COVID-19 pandemic (1) Türkiye is among the OECD nations that attract a substantial share of students from lower-income regions, mainly Central Asia, the Middle East, and Africa, leading to increasingly diverse linguistic and cultural student populations at its universities (2, 3).

This global movement is significant in nursing education. The International Council of Nurses’ (ICN) “Recover to Rebuild” report highlights a significant worldwide nursing shortage and underscores that many health systems depend on expanding nursing education capacity, international mobility, and cross-border training to stabilize and rebuild the workforce (ICN, 2021). Consequently, the education and training of international nursing students has become a critical component of preparing a competent and sustainable healthcare workforce (4–6).

As international students enter undergraduate nursing programs, they encounter educational environments shaped by local pedagogical traditions and healthcare expectations. Previous studies have documented challenges related to language proficiency, classroom communication norms, cultural differences in instructor student interactions, and adaptation to unfamiliar educational systems (7–10). These challenges are particularly salient during psychomotor skill learning, where students are required to integrate theoretical knowledge, procedural accuracy, reflective reasoning, and emerging professional identity (11, 12).

Simulation-based education is a cornerstone of contemporary health professions education, offering structured demonstrations, scaffolded practice, formative feedback, and repeated performance opportunities that support psychomotor skill development across diverse learner populations (13, 14). However, for international nursing students, early laboratory experiences may also generate cognitive overload, emotional stress, and uncertainty, especially when learning occurs in a second language or unfamiliar cultural context (2, 3). While previous research has explored international students’ academic and clinical adaptation more broadly, the developmental learning processes that unfold during early simulation-based psychomotor training remain insufficiently understood (15, 16).

Notably, existing literature often addresses international students’ difficulties in a fragmented manner, focusing separately on language barriers, cultural adjustment, or technical skill acquisition (10, 14, 17). Less attention has been paid to how cognitive, emotional, experiential, and reflective processes interact as students’ progress from initial uncertainty toward confidence, procedural competence, and early professional identity formation during psychomotor learning (18). This gap highlights the need for a process-oriented perspective that captures how learners interpret, navigate, and make meaning of early laboratory experiences within health education.

Psychomotor skill development in nursing education is inherently multidimensional, involving the integration of cognition, practice, reflection, emotional regulation, and identity development. To examine these interconnected processes, two complementary theoretical frameworks are particularly informative: Kolb’s Experiential Learning Model (ELM) and Mezirow’s Transformative Learning Theory (TLT). Kolb conceptualizes learning as a cyclical process encompassing concrete experience, reflective observation, abstract conceptualization, and active experimentation, a structure that closely aligns with simulation-based skills training (19). In contrast, Mezirow’s theory emphasizes how disorienting experiences stimulate reflection, reassessment of assumptions, and perspective transformation, processes frequently encountered by international students facing linguistic, cultural, and performance-related challenges in the skills laboratory (20, 21).

Although both frameworks have been applied in nursing and health professions education, their integrated use to explain early psychomotor learning experiences in culturally diverse simulation settings remains limited (7, 17, 22). Combining experiential and transformative perspectives offers a more comprehensive understanding of how technical skill acquisition intersects with emotional adaptation, reflective meaning-making, and early professional identity formation in health education contexts.

### 1.1 Rationale for an integrated framework and theoretical contribution of TELM

Although Kolb’s ELM and Mezirow’s TLT are well-established in educational research, each framework has specific limitations when applied to simulation-based psychomotor skill learning among international nursing students. Kolb’s model offers a robust cyclical structure for understanding experiential learning (concrete experience, reflective observation, abstract conceptualization, and active experimentation); however, it does not account for the emotional disruption, identity negotiation, or cultural linguistic barriers that characterize international students’ early laboratory encounters. The model implicitly assumes a relatively homogeneous learner profile progressing through stages in a sequential, culturally neutral manner, without addressing how disorienting experiences may fundamentally alter learners’ frames of reference.

Conversely, Mezirow’s TLT offers a powerful lens for understanding how critical reflection on disorienting experiences can lead to perspective transformation and identity development. However, this framework has been applied primarily in clinical, workplace, or post-licensure educational contexts and does not provide a structured mechanism for explaining how hands-on procedural practice contributes to transformative processes. Nor does it explicitly address psychomotor skill acquisition as a vehicle for meaning-making and professional growth.

The Transformative Experiential Learning Model (TELM) proposed in this study bridges complementary gaps. Its distinctive contribution lies in three key areas: first, it demonstrates that experiential learning cycles in preclinical simulation environments create the preconditions for transformation, positioning the skills laboratory as a critical early site where transformation begins earlier than typically recognized in the literature. Second, TELM conceptualizes technical skill acquisition and the formation of an emerging professional identity as concurrent, mutually reinforcing processes rather than as sequential or independent outcomes. Third, it explicitly integrates cultural linguistic diversity as a contextual catalyst for transformation, recognizing that the challenges international students face (e.g., language barriers, unfamiliar pedagogical norms, performance anxiety in a second language) function not merely as obstacles but as disorienting dilemmas that activate deeper reflective and transformative learning. A comparison of these three frameworks is presented in Table 1.

**Table 1:** Comparison of Kolb’s experiential learning model, Mezirow’s transformative learning theory, and the proposed transformative experiential learning model across key theoretical dimensions.

Dimension	Kolb’s experiential learning model (ELM)	Mezirow’s transformative learning theory (TLT)	Transformative experiential learning model (TELM)
Epistemological orientation	Pragmatic constructivism emphasizes learning through experience and reflection	Critical constructivism emphasizing transformation of meaning perspectives through critical reflection	Contextual constructivism integrating experiential learning with reflective meaning-making in culturally diverse simulation environments
Primary focus	Experiential learning cycle	Perspective transformation through critical reflection	Developmental integration of experiential skill learning and early professional identity formation
Core mechanism	Concrete experience → Reflection → Conceptualization → Experimentation	Disorienting dilemma → Critical reflection → Perspective transformation	Experiential cycles function as catalysts for early transformative and identity-oriented development during psychomotor skill acquisition
Treatment of emotion	Implicit emotional responses embedded within learning experiences	Central; emotional disruption initiates critical reflection and transformation	Emotional disruption (e.g., anxiety, linguistic insecurity, cultural uncertainty) acts as a trigger for reflective engagement within skill learning
View of psychomotor skill learning	Procedural and cognitive skill development through repeated practice and reflection	Not specifically focused on procedural or psychomotor learning	Psychomotor learning is conceptualized as embodied meaning-making linked to identity formation and professional role internalization
Cultural–Linguistic context	Learning cycle is assumed to be culturally neutral	Funding The author(s) declare that no financial support was received for the research and/or publication of this article. Conflict of Interest Statement The authors declare no competing interests. Acknowledges social context, but is not specific to multilingual learning environments	Cultural and linguistic challenges are interpreted as catalysts for reflective engagement and developmental transformation in international learning contexts
Role of simulation-based education	Compatible with experiential learning cycles but not explicitly linked to identity development	Rarely applied to simulation-based or preclinical educational environments	Simulation laboratory conceptualized as a developmental site where experiential practice, reflective dialogue, and identity negotiation intersect
Temporal positioning of transformation	Transformation is not explicitly addressed	Typically conceptualized in adult learning or professional practice contexts	Repositions transformative processes as emerging during preclinical simulation-based psychomotor learning
Professional identity formation	Not explicitly addressed	Identity transformation emerges through perspective transformation	Professional identity development and procedural competence are conceptualized as concurrent and mutually reinforcing processes
Theoretical scope	Process-oriented learning theory explains experiential learning cycles	Adult learning theory explains perspective transformation	Context-bound conceptual framework integrating experiential, transformative, and cross-cultural dimensions of simulation-based learning

Therefore, this study aims to explore international nursing students’ experiences of psychomotor skill development during simulation-based laboratory training and to examine the learning processes underlying these experiences through an integrated experiential–transformative lens.

## 2 Methods

### 2.1 Study design

This study employed an interpretive qualitative design informed by experiential and transformative learning theories. The study adopted a constructivist epistemological stance, acknowledging that participants’ learning experiences are shaped by subjective meaning-making processes situated within specific cultural, linguistic, and educational contexts. This interpretive orientation guided the research team’s engagement with participants’ accounts, focusing not only on what students experienced but also on how they interpreted, negotiated, and made meaning of those experiences. Accordingly, the TELM developed from this analysis is positioned as a context-bound conceptual framework rather than a mid-range theory; it offers an interpretive lens for understanding how experiential and transformative learning processes intersect in preclinical simulation settings among international students, while requiring further empirical testing across diverse populations and contexts. The study was designed to explore how international undergraduate nursing students experience psychomotor skill learning in a simulation-based laboratory environment. Kolb’s Experiential Learning Model and Mezirow’s Transformative Learning Theory were used as sensitizing frameworks to inform the development of the interview guide and orient the analytic focus toward experiential, cognitive, and emotional aspects of learning, without imposing predetermined categories. The study was conducted and reported in accordance with the Consolidated Criteria for Reporting Qualitative Research (23).

### 2.2 Sample and setting

This study was conducted at the Faculty of Health Sciences of a nonprofit foundation university located in Istanbul, Türkiye. The institution offers a nationally accredited undergraduate nursing program aligned with the standards set by the National Nursing Education Accreditation Board. The university hosts a culturally diverse student population, with a significant proportion of international students originating primarily from Central Asia and the Middle East, whose linguistic, cultural, and educational backgrounds differ considerably from those of Türkiye. This diversity creates a unique learning environment in which international students navigate both academic and sociocultural transitions. All nursing courses, including those involving psychomotor skill acquisition, are delivered in Turkish, the program’s official language of instruction. Psychomotor learning is supported through structured simulation-based laboratory sessions that incorporate visual instructional materials, instructor-led demonstrations, hands-on practice stations, and iterative feedback cycles. A total of 20 international undergraduate nursing students participated in the study. Participants were selected using maximum variation purposive sampling to ensure representation across a wide range of demographic and educational characteristics, including cultural background, length of residence in Türkiye, academic standing, and prior exposure to laboratory based learning. The following were the inclusion criteria: (1) international student status, (2) enrollment in the Turkish-medium undergraduate nursing program, (3) completion of at least one laboratory-based psychomotor skills course, and (4) voluntary agreement to participate in a comprehensive interview. Exclusion criteria included the following: (1) international students without prior completion of a laboratory-based psychomotor skills course, (2) inability to participate in a verbal interview owing to language barriers beyond the support available in Turkish or English, and (3) discomfort with audio recording or refusal to provide verbal consent.

### 2.3 Data collection

Data were gathered through semi-structured individual interviews to gain a detailed understanding of international nursing students’ experiences with psychomotor skill learning in the simulation laboratory (See supplementary materials for interview questions). The interview guide was created using Kolb’s Experiential Learning Model and Mezirow’s Transformative Learning Theory as guiding concepts, emphasizing the importance of experiential involvement, reflective thinking, emotional reactions, and meaning making in learning. These frameworks shaped the overall direction of the questions but did not limit or predetermine participant responses.

Data collection took place from February to April 2025. All interviews were conducted face-to-face in a private room on campus to ensure confidentiality and reduce distractions. Interviews were conducted by three faculty members holding doctoral degrees with research and practice experience in nursing education and skills training. None of the interviewers had any instructional or evaluative relationship with the participants, ensuring that grade-related dynamics did not influence participants’ responses. Each interview lasted about 30–45 min and was audio recorded with participants’ informed consent (See Supplementary file for interview questions). To capture contextual details, including nonverbal cues, field notes were taken during and immediately after each interview. Although all participants attended a Turkish medium program, they had different levels of Turkish proficiency. To minimize communication barriers and allow more accurate expression of experiences, participants could respond in English at any point during the interview. When participants switched languages, the transcript kept the original segments in Turkish or English. All recordings were transcribed verbatim, pseudonyms were assigned, and transcripts were checked for accuracy before analysis. This method supported the credibility and depth of multilingual data in line with COREQ standards. Regarding multilingual data handling, interviews were not translated into a single language; transcripts preserved participants’ original expressions in both Turkish and English to maintain semantic authenticity. Coding was conducted primarily in Turkish, the language in which the majority of the interview content was produced, while English segments were coded in English. To ensure meaning equivalence, two bilingual members of the research team independently reviewed coded segments, comparing interpretations across languages and discussing discrepancies until consensus was reached. This approach minimized translation-related meaning loss and preserved the linguistic nuances of participants’ accounts.

### 2.4 Data analysis

Data were analyzed using MAXQDA Analytics Pro 2024 (VERBI Software, Berlin, Germany), employing a hybrid thematic analysis that combined inductive and deductive strategies. This approach aligns with recommendations for rigorous thematic analysis that balances data driven coding with conceptual interpretation (24). The analytic process was guided by the experiential and transformative learning concepts previously described, which served as sensitizing lenses that directed attention to cognitive, emotional, and experiential aspects of learning while preserving openness to themes emerging directly from the data (25, 26). The analysis proceeded in three main stages:

*Stage 1: Familiarization and inductive open coding:* The analytic process began with repeated readings of all transcripts to establish immersion in the data. Each transcript was coded line by line, and inductive codes were generated to capture participants’ descriptions of experiential challenges, emotional responses, facilitators, cultural and linguistic barriers, reflective processes, and skill-related learning experiences. Codes remained closely grounded in participants’ own words to reflect their perspectives authentically (24, 27).

*Stage 2: Theory-informed coding:* In the second stage, deductive coding drew on concepts from Kolb’s and Mezirow’s theoretical frameworks. Inductive codes were compared with constructs such as experiential engagement, reflective observation, meaning making, and confidence development. These sensitizing concepts were applied flexibly, acknowledging that participants’ accounts were nonlinear and often overlapped multiple experiential and reflective dimensions (25, 26).

*Stage 3: Integration, synthesis, and conceptual mapping:* Inductive and theory-informed codes were then compared and clustered using MAXQDA’s visualization tools, including code co-occurrence models, code maps, and matrix analyses. These analytic outputs supported the integration of codes by illustrating relationships across experiential, reflective, and emotional dimensions of learning. This process facilitated the organization of a coherent thematic structure, resulting in four themes and 14 subthemes, which together offered a comprehensive representation of international students’ psychomotor skill learning experiences within a culturally diverse simulation laboratory. This iterative process across the three stages resulted in a finalized coding scheme, which was applied consistently across all transcripts.

Importantly, the construction of the TELM was driven by this iterative analytic process rather than by *a priori* theoretical imposition. During inductive open coding, recurring patterns emerged that neither Kolb’s nor Mezirow’s framework alone could fully capture, for example, students’ descriptions of concurrent skill acquisition and identity shifts, or the role of cultural–linguistic barriers as catalysts for deeper reflection. These inductively identified patterns were then examined through theory-informed coding, revealing points of convergence and divergence with existing constructs. The resulting TELM stages were thus empirically grounded in participant narratives and refined through theoretical dialogue, ensuring that the model reflects the data rather than merely confirming pre-existing frameworks. A detailed mapping of themes, subthemes, codes, TELM components, and illustrative student quotations is provided in Table 2.

**Table 2:** Mapping of themes, subthemes, codes, and student quotations onto the transformative experiential learning model (TELM).

Main category	Subcategory	Codes	TELM components	Student quotations
Factors making learning difficult	Use of technological educational platforms	- Difficulty with English videos - Lack of subtitles	Disorienting dilemma	“*The technologies used in education were different from the system in Iran; in the first few weeks, I was confused about which button to click.*” (P02) “*The platform was entirely in Turkish. I clicked in the wrong places because I did not understand some of the words.*” (P09)
	Performance anxiety	- Feeling nervous during examinations - Tension under supervision	Self-examination	“*I got very excited when I was left alone while watching the teacher that I mixed up the steps.*” (P09) “*As someone who is older and has a speech impediment, I remained shy. I fell silent for fear of using the wrong word.*” (P15) “*My hands trembled while my friends were watching, and I could not perform what I normally know how to perform.*” (P04)
	Cultural differences	- Difficulty adapting to different teaching methods - Unfamiliarity with the practice culture	Disorienting dilemma	“*Afraid patients would not trust me because of my name.*” (P06) “*In my own country, it was normal to ask the teacher questions; however, here, it was difficult for me to ask questions in front of everyone.*” (P12)
	Difficulty with medical terminology	- Difficulty adapting to Latin terms - Confusion with medical concepts	Critical assessment of assumptions	“*Regarding scientific terms, I have no problem speaking; however, academic Turkish can be difficult.*” (P01) “*Latin equivalents are slightly difficult; I hesitate.*” (P04)
	Language deficiency	- Difficulty expressing oneself - Inability to use Turkish at an academic level	Critical assessment of assumptions	“*I understand what the teachers say; however, I find it difficult to explain in Turkish why I am taking this step.*” (P11) “*Sometimes, I know the answer clearly in my own language; however, I remain silent because I cannot express it in Turkish.*” (P15)
Facilitators of learning	Use of visual educational materials	- Support through PDF and video materials - Preference for Turkish content	Concrete experience, reflective observation	“*He had uploaded a video of the application; when I watched that video, I understood everything at once.*” (P20) “*When I watched the videos along with the PDF, the movements stick in my mind.*” (P11) “*Without the pictures and videos, it would be difficult for me to understand some of the steps from the words alone.*” (P08)
	Effective feedback	- Immediate corrective feedback - Positive reinforcement	Critical assessment of assumptions, recognition of shared discontent	“*When they directly point out my mistakes, it motivates me. When they say, ‘You can do better, try again,’ I go home and work until I correct that mistake.*” (P04) “*When they clearly say, ‘Correct this step, do it this way,’ I correct it immediately; seeing and instantly correcting the mistake accelerated my learning.*” (P11) “*When the teacher stands over me and tells me step by step what I did wrong, I correct it right then and learn the right way.*” (P07)
	Instructor guidance	- Step-by-step guidance - Observing the instructor as a role model	Planning a course of action, acquiring knowledge and skills	“*I realized how significant it is to learn in a supportive environment. When you are not afraid of making mistakes, the learning process becomes enjoyable.*” (P15) “*The fact that they constantly observe and intervene when necessary provides me confidence and helps me improve.*” (P02) “*Sometimes, I hesitate when I try to do things on my own; however, when the teacher guides me step by step, I learn much faster*.” (P20)
	Peer support	- Practicing with peers - Learning through peer explanations	Recognition of shared discontent, trying new roles	“*My Turkish friends explain things; I understand more easily.*” (P01) “*I ask my friends questions… then it becomes clear.*” (P12)
Psychomotor learning pathways	Supportive role of educational materials	- Recalling steps using laboratory manuals - Visual learning through video	Acquiring knowledge and skills	“*Before entering the class, our checklists and videos are shared. I always watch the videos beforehand; that way, I have a clear idea of which steps to take and in what order.*” (P06) “*The steps explained using diagrams and pictures stay clearer in my mind; therefore, I make fewer mistakes when applying them.*” (P12)
	Learning by doing	- Learning by repeating the skill - Learning from mistakes	Active experimentation, building competence	“*The teacher explains first; subsequently, we repeat it immediately. Seeing and doing at the same time fixes the information in my mind.*” (P18) “*I made several mistakes on my first attempt; however, as I repeated it, my hand got faster, and now the steps are automatic.*” (P07)
	Observation	- Learning by observing the practice - Reinforcement through role modeling	Recognition of shared discontent, reflective observation	“*Our teachers demonstrated live and then had us repeat it, enabling me to learn by seeing.*” (P20) “*Watching my friends practice, I realized my shortcomings; therefore, when it was my turn, I performed it more correctly.*” (P14)
Cognitive and emotional transformation	Transition to professional identity	- Developing professional self - Awareness of roles - Feeling a sense of belonging to the profession	Exploration of new roles, reintegration	“*When I first arrived, I felt very inadequate; however, now, I realize I think like a nurse.*” (P07) “*As I gained experience, my approach changed. It is not just about performing procedures; understanding how they feel is also important.*” (P12) “*I now believe I can do this job. I see myself not just as a student, but as a nurse.*” (P03)
	Developing self-confidence	- Increased self-confidence - Ability to express oneself - Motivation for self-improvement	Building competence and self-confidence, trying new roles	“*When I first arrived, I was nervous; now, I have gotten used to both the teachers and my classmates, and I feel at ease. My confidence grew as I learned and applied the skill steps.*” (P20) “*In the first few weeks, I did not know anyone, and I was afraid to ask questions. Now, when I enter the laboratory, I am the one who says, ‘Let me do it.’ I am not afraid of making mistakes.*” (P03)

Reflexive memos, field notes, and team discussions were integrated throughout the analytic process to enhance dependability, confirmability, and theoretical sensitivity (24). A research team with expertise in nursing education, psychomotor skill instruction, and qualitative research conducted all interviews and analyses (23).

#### 2.4.1 Rigor and reflexivity

Rigor in this qualitative study was ensured through strategies addressing credibility, dependability, confirmability, and transferability (24). An audit trail documenting all methodological decisions, coding revisions, and analytic memos established transparency throughout the research process (23, 27).

Credibility was enhanced through several complementary methods. Member checking allowed participants to confirm the accuracy of initial interpretations, while peer debriefing sessions helped the research team carefully examine emerging themes and identify potential blind spots. Triangulation of interview transcripts, field notes, and reflexive memos further validated the authenticity of the findings (24).

To improve dependability, two researchers independently coded 20% of the transcripts. Discrepancies were discussed until consensus was reached, helping to refine the codebook and establish intercoder reliability (28).

Confirmability was enhanced by maintaining detailed reflexive and analytic memos that documented interpretive decisions and ensured that findings were rooted in participants’ accounts rather than researcher assumptions (25). Reflexive engagement was central throughout the study. The research team included five members: two with doctorates in nursing education and three in fundamentals of nursing, including two professors with extensive experience in qualitative inquiry and cross-cultural educational settings. Because they were partly insider faculty members at the same institution, the researchers remained alert to potential biases. Reflexive journals documented evolving interpretations, emotional reactions, and assumptions about participants’ language challenges, cultural subtleties, and classroom power dynamics. For example, moments of participant silence, initially seen as disengagement, were later recognized as hesitation due to linguistic insecurity, leading to more supportive and adaptive interview strategies.

Transferability was addressed by providing a detailed description of the institutional context, student population, curriculum structure, and simulation laboratory environment, allowing readers to evaluate the relevance of the findings to other multicultural nursing programs (23).

By systematically combining strategies that enhance rigor with continuous reflexive practice, the study ensured methodological trustworthiness and offered contextually grounded insights into international nursing students’ psychomotor learning experiences.

### 2.5 Ethical considerations

This study received ethical approval from the Medical Research Ethics Committee of Acıbadem Mehmet Ali Aydınlar University (Approval No: 2025-01/52; Date: January 09, 2025). Prior to data collection, all participants were provided with detailed information about the study’s aims, procedures, potential risks, and their rights as voluntary participants. Given the multilingual and cross-cultural composition of the student group, written informed consent was obtained and audio-recorded in accordance with institutional guidelines and the approved protocol. Participants were explicitly informed that participation was voluntary, that they could decline to answer any question or withdraw from the study at any stage without negative consequences, and that their decision would not influence their academic evaluation or standing within the program. To safeguard confidentiality and anonymity, pseudonyms were assigned during transcription, and all audio files and transcripts were stored on encrypted, password-protected devices with access restricted to the research team. Identifying details were removed or anonymized to ensure that participants could not be recognized in the dissemination of findings. This research was conducted in accordance with the ethical principles of the Declaration of Helsinki and complied with national regulations governing research involving human participants in Türkiye. All procedures adhered to internationally accepted ethical standards for qualitative research, particularly for studies involving culturally and linguistically diverse student populations.

## 3 Results

This study involved 20 international students; all enrolled in a nursing degree program. Of the participants, 85% (*n* = 17), 10% (*n* = 2), and 5% (*n* = 1) were from Turkmenistan, Iran, and Afghanistan, respectively. Regarding gender distribution, 85% (*n* = 17) were female and 15% (*n* = 3) were male. The participants’ ages ranged from 18 to 33 years, with a mean age of 22.85 ± 4.47 years. Most participants had been in Turkey for at least 1 year (Table 3).

**Table 3:** Sociodemographic characteristics (*n* = 20).

Participants	Age (years)	Sex	Country	Length of stay in Turkey
P01	20	Female	Turkmenistan	11 months
P02	24	Male	Iran	3 years
P03	19	Female	Turkmenistan	1 years
P04	33	Female	Turkmenistan	1.5 years
P05	25	Female	Afghanistan	4 years
P06	19	Female	Iran	3 years
P07	25	Female	Turkmenistan	9 months
P08	20	Male	Turkmenistan	2 years
P09	19	Female	Turkmenistan	7 years
P10	22	Female	Turkmenistan	1.5 years
P11	20	Female	Turkmenistan	3 years
P12	18	Male	Turkmenistan	1 years
P13	20	Female	Turkmenistan	2 years
P14	28	Female	Turkmenistan	5 years
P15	30	Female	Turkmenistan	1.5 years
P16	20	Female	Turkmenistan	2 years
P17	30	Female	Turkmenistan	1 years
P18	22	Female	Turkmenistan	3 years
P19	25	Female	Turkmenistan	3 years
P20	18	Female	Turkmenistan	8 months

**Figure 1:**
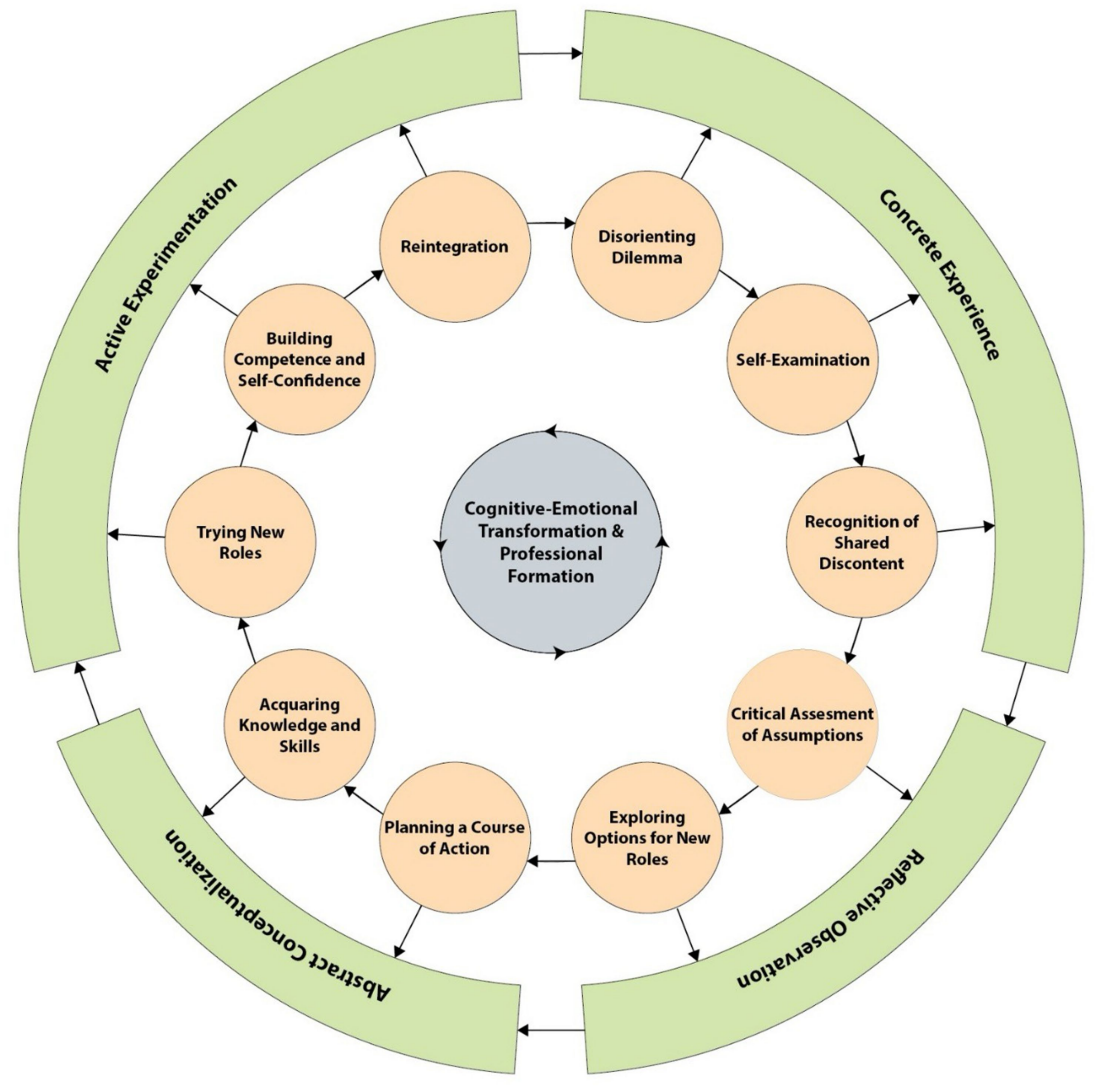
Transformative–Experiential Learning Model (TELM): integration of experiential and transformative learning in psychomotor skill acquisition among international nursing students. The model presented in this figure was developed by the authors based on the findings of the current study and is published for the first time in this article.

Inductive thematic analysis identified four main themes, 14 subthemes, and 30 codes that collectively reflected students’ learning experiences in psychomotor skills training. During analysis, students’ narratives were systematically examined through the complementary perspectives of Mezirow’s Transformative Learning Theory and Kolb’s Experiential Learning Cycle, enabling the research team to interpret not only what students experienced but also how they progressed through cognitive, emotional, and behavioral phases of learning. By mapping participant quotations and emergent codes onto the sequential stages of these two foundational frameworks, a unified developmental trajectory was identified that captured students’ movement from initial disorientation to reflection, conceptual understanding, active experimentation, and ultimately transformation. This integrated analysis resulted in the development of the Transformative Experiential Learning Model (TELM), which combines key elements of Mezirow and Kolb to explain students’ evolving learning pathways (Figure 1). Accordingly, the Findings section is organized around TELM’s components, providing a structured interpretation of how international nursing students navigated and made meaning of their psychomotor learning experiences (Table 2). Table 2 provides a comprehensive mapping of all four themes, 14 subthemes, and 14 codes onto the corresponding TELM stages, accompanied by illustrative student quotations that demonstrate the empirical grounding of each model component. In synthesizing the thematic findings into TELM stages, the research team observed that the four themes reflected a progressive developmental trajectory in students’ learning experiences. Difficulties encountered during early skill acquisition corresponded to the initial stages of disorientation and self-examination, while facilitating factors aligned with reflective reinterpretation and role exploration. The psychomotor learning pathways described by participants corresponded to the experiential phases of conceptualization, planning, and active experimentation, whereas the theme of cognitive and emotional transformation captured the later integrative stages of competence development and emerging professional identity. This thematic-to-stage mapping was iteratively refined through team discussions to ensure that the model accurately represented the developmental patterns evident across participants’ narratives.

### 3.1 Theme 1: Factors making learning difficult

International nursing students’ initial experiences with psychomotor skills training were shaped by a series of interconnected challenges that significantly impacted their entry into the learning process. These early difficulties reflect the initial stages of the TELM, starting with Concrete Experience, where learners encounter new expectations and unfamiliar demands. For many students, the first obstacles appeared as they tried to navigate digital learning platforms and technology-based instructional systems. Interfaces in Turkish, unfamiliar menu structures, and limited prior exposure to similar platforms confused and hindered their initial interactions with course materials. These technological challenges represented a clear disorienting dilemma, forcing students to realize that their previous learning strategies were inadequate in this new environment. As one student described: “*The technologies used in education were different from the system in Iran; in the first few weeks, I was confused about which button to click*” *(P02).*

Alongside technological barriers, students faced significant performance anxiety, especially during supervised procedures and peer-observed demonstrations. The pressure to perform complex skills in a new language, combined with the fear of making visible mistakes, triggered emotional and physical reactions such as trembling, hesitation, or freezing. These responses reflect TELM’s phase of self-examination, where learners begin to confront the emotional aspects of their learning challenges and recognize gaps in confidence, communication, and preparedness. The anxiety was often worse for students with limited language skills or those who viewed themselves as different from their peers because of age or cultural background. *For example, one participant noted: “My hands trembled while my friends were watching, and I could not perform what I normally know how to perform” (P04).*

Cultural differences further contributed to the initial feeling of disruption. Adjusting to new educational norms, such as more direct questioning styles, different expectations about student participation, and diverse approaches to instructor student interactions, challenged students’ established beliefs about appropriate classroom behavior. These mismatches amplified the disorienting dilemma by making students realize that their familiar cultural and educational patterns did not easily transfer to the new environment. As a result, they were forced to reexamine previous assumptions about how learning and communication should happen. One student illustrated this shift: *“In my own country, it was normal to ask the teacher questions; however, here, it was difficult for me to ask questions in front of everyone”* (P12). This hesitation appeared to stem not from a lack of willingness but from a perceived difference in classroom communication norms; the student felt that the social dynamics of the new learning environment, including language insecurity and unfamiliarity with expected participation styles, inhibited behaviors that had been natural in a familiar cultural context.

Linguistic challenges added an extra layer of complexity to the learning process. Students often struggled with medical terminology, especially Latin-derived anatomical terms or procedural vocabulary that differed from conversational Turkish. These difficulties affected their understanding and created uncertainty when they were expected to explain clinical rationales verbally. Such linguistic gaps led to a critical evaluation of assumptions, as students recognized a gap between their everyday language skills and the academic–clinical communication expected in the nursing program. Beyond terminology, broader language issues, including problems with academic Turkish, difficulty describing procedural steps, or discomfort speaking in front of others, further limited participation and reduced students’ willingness to engage in laboratory activities actively. As one student explained: *“I understand what the teachers say; however, I find it difficult to explain in Turkish why I am taking this step”* (P11). Another noted: *“Sometimes, I know the answer clearly in my own language; however, I remain silent because I cannot express it in Turkish”* (P15).

Although these challenges were individual, students gradually realized that their peers faced similar struggles. Realizing that common issues like confusion with digital tools, anxiety, cultural adjustment difficulties, and language barriers were shared helped lessen feelings of isolation. It marked TELM’s Recognition of Shared Discontent stage. This shared awareness changed the view of these obstacles from personal failures to standard parts of adapting to a new educational environment. For many, this realization was a turning point, making the learning environment feel more manageable and less intimidating.

Overall, the challenges students faced during their initial engagement in the psychomotor skills lab show how the earliest stage of learning is affected by unfamiliar experiences, emotional distress, language barriers, and cultural adjustments. These interconnected experiences trigger the foundational phases of TELM, creating the conditions for the reflective, conceptual, and experimental learning processes that develop in later stages of their transformative learning journeys.

### 3.2 Theme 2: Factors facilitating learning

As international nursing students moved beyond the initial challenges of laboratory-based psychomotor skills training, a range of pedagogical, social, and material supports played a crucial role in helping them progress through the TELM stages. These supports align with Reflective Observation, where learners begin to understand their early experiences by observing others, seeking clarification, and integrating structured input. Visual educational materials, such as videos, diagrams, and step-by-step guides, enabled students to revisit procedures, enhance their memory, and compare their understanding with correct demonstrations. This reflective engagement with visual cues helped decrease uncertainty and provided a mental anchor that bridged linguistic barriers. As one student reflected: *“Without the pictures and videos, it would be difficult for me to understand some of the steps from the words alone”* (P08).

As students actively reflected on their performances, effective instructor feedback proved to be another strong facilitator. Immediate corrective input helped learners identify discrepancies between their intentions and what was needed, aligning with TELM’s stage of Critical Assessment of Assumptions. Through this corrective and constructive process, students recognized incorrect assumptions about procedural steps, improved their understanding of technique, and gained confidence in their ability to self-correct. The timeliness and clarity of feedback prevented repeated errors and accelerated skill development, boosting learners’ motivation to keep going. One student described: *“When they directly point out my mistakes, it motivates me. When they say, ‘You can do better, try again,’ I go home and work until I correct that mistake”* (P04).

Instructor guidance further reinforced this adaptive process by providing students with tangible models to observe and imitate. Educators’ step-by-step demonstrations, encouraging communication, and interventions during practice helped students gain a clearer understanding of expectations and procedural sequences. This form of guided reflection acts as a bridge between observational learning and the development of more autonomous reasoning about practice. Through instructor modeling, students became more comfortable recognizing their learning needs, reducing the fear of judgment and enabling deeper engagement with practice activities. *As one student noted: “Sometimes, I hesitate when I try to do things on my own; however, when the teacher guides me step by step, I learn much faster” (P20).*

Peer support also played a key role in strengthening students’ ability to engage in reflective learning. Working with classmates, especially Turkish peers, helped clarify misunderstandings, verify procedural steps, and encourage open discussion of challenges. Knowing that peers faced similar uncertainties provided reassurance and fostered a socially supportive environment ideal for collaborative understanding. Peer explanations improved linguistic and conceptual clarity, helping co-create knowledge and guiding students to reach TELM’s Exploring Options for New Roles stage. At this stage, learners start experimenting with new ways of participating, asking questions more actively, practicing more often, and taking more initiative in group tasks. *One student simply stated: “My Turkish friends explain things; I understand more easily” (P01).*

### 3.3 Theme 3: Psychomotor learning pathways

International nursing students described acquiring psychomotor skills as a gradual and intentional process influenced by structured preparation, cognitive organization, and increasingly confident practice. Their descriptions closely match the mid-cycle stages of the TELM. Students initially engaged in Abstract Conceptualization, using instructional videos, diagrams, checklists, and written explanations to mentally rehearse and understand procedures before entering the laboratory. These resources helped them build a mental map of each skill, visualize the correct sequence of actions, and reduce uncertainty about what to expect during practice. Building on this cognitive foundation, learners moved into Planning a Course of Action, using information from visual materials and instructor guidance to plan their approach, anticipate complex steps, and strategize ways to correct common errors. This planning phase increased their sense of preparedness and decreased hesitation during hands-on practice. Through repeated exposure and structured preparation, students progressed to Acquiring Knowledge and Skills, where they integrated conceptual understanding with practical strategies, deepened their comprehension of rationale-based actions, and showed increased accuracy in performing procedure steps. As learners repeatedly practiced skills, corrected mistakes, and refined techniques through observation of instructors and peers, they entered the Active Experimentation stage. This phase allowed them to turn planned actions into physical performance, test their understanding through real practice, and gradually develop technical fluency and confidence. Observational learning further supported this growth, enabling students to mimic proper techniques and identify performance gaps before attempting skills independently. Together, these interconnected processes demonstrate how students’ psychomotor learning develops through a cyclical process of conceptual preparation, strategic planning, skill acquisition, and increasingly confident practice, ultimately fostering procedural competence in the laboratory environment. One student explained: *“Before entering the class, our checklists and videos are shared. I always watch the videos beforehand; that way, I have a clear idea of which steps to take and in what order” (P06).* Another described the role of repetition: *“I made several mistakes on my first attempt; however, as I repeated it, my hand got faster, and now the steps are automatic” (P07).* Observational learning was also valued: *“Watching my friends practice, I realized my shortcomings; therefore, when it was my turn, I performed it more correctly” (P14).*

### 3.4 Theme 4: Cognitive and emotional transformation

As students progressed through laboratory-based psychomotor skills training, they experienced a significant cognitive and emotional transformation that extended beyond mere technical skill learning. This change aligns with the later stages of the TELM, where learners actively test their new abilities, assume new roles, and gradually reshape their professional identities. Through repeated practice and increased familiarity with procedures, students began engaging in Active Experimentation, applying what they learned more independently and incorporating feedback with greater confidence. This active participation created opportunities to explore new roles, as learners shifted from passive observers to proactive contributors who volunteered to demonstrate skills, asked more questions, and took initiative during practice. Over time, these experiences boosted their sense of control and self-efficacy, advancing TELM’s stage of Building Competence and Self-Confidence. Students reported that initial feelings of insecurity, hesitation, and inadequacy diminished as their procedural skills improved and confidence grew. They described feeling more comfortable with instructors and peers, less afraid of making mistakes, and more motivated to improve their skills. More profound cognitive changes accompanied this ever-increasing confidence: learners started viewing skill performance not just as a technical task but as an integrated nursing activity that requires empathy, ethical awareness, and professional judgment. Ultimately, these ongoing experiences led to Reintegration, during which students reported starting to “think like nurses” rather than simply completing tasks as novices. They felt a stronger connection to the profession, recognized their responsibilities as caregivers, and visualized themselves more clearly as emerging healthcare professionals. As one student reflected: *“When I first arrived, I felt very inadequate; however, now, I realize I think like a nurse”* (P07). Another described this transformation in terms of confidence and agency*: “In the first few weeks, I did not know anyone, and I was afraid to ask questions. Now, when I enter the laboratory, I am the one who says, ‘Let me do it.’ I am not afraid of making mistakes”* (P03). This overall process demonstrates how continuous practice, emotional resilience, and reflective engagement with learning tasks collectively support students’ cognitive and emotional development, enhancing their growth into confident, integrated nursing professionals.

Overall, the four themes collectively demonstrate how international nursing students’ learning developed as a layered, evolving process influenced by cognitive, emotional, social, and experiential factors. Instead of depicting isolated challenges or facilitators, the findings show a continuous movement through the phases of the TELM, starting with disorientation and progressing toward reflection, conceptual reorganization, and more confident engagement with practice. This developmental progression highlights how students gradually absorbed new knowledge, navigated cultural and linguistic complexities, restructured previous assumptions, and experimented with new ways of acting in the laboratory setting. The synthesis presented in Figure 1 captures this trajectory, illustrating how the components of TELM intersect with students’ lived experiences to support both skill development and deeper professional growth. These insights not only clarify the mechanisms underlying students’ learning pathways but also provide a conceptual link to the discussion section, where the educational implications of these transformative processes are examined in greater depth.

## 4 Discussion

This study explored international nursing students’ psychomotor learning experiences using Mezirow’s Transformative Learning Theory and Kolb’s Experiential Learning Cycle. The findings show that students’ development in the skills lab follows a multidimensional path that includes emotional upheaval, reflective interpretation, conceptual restructuring, and early professional identity formation. While earlier research mainly viewed transformative learning as primarily happening in clinical settings, this study suggests that early transformative processes and emerging identity development begin much earlier during structured lab practice. To explain this process, we created the TELM, which combines experiential mechanisms with identity changes and addresses a theoretical gap by showing how procedural competence and professional identity develop together in preclinical settings. Specifically, TELM functions as an interpretive conceptual framework that traces a developmental trajectory from initial disorientation through reflective engagement to emerging professional identity during simulation-based psychomotor skill learning. Rather than proposing a broadly generalizable theory, the framework provides an empirically grounded structure for understanding how experiential, reflective, and early transformative learning processes intersect within a specific preclinical context and may guide future research across diverse educational settings. These insights emphasize a key point: early transformative processes in nursing education may start much earlier than often thought, prompting a reevaluation of how early laboratory learning is understood and supported.

A key finding was that anxiety, uncertainty, and feelings of inadequacy characterized students’ initial experiences with psychomotor skills. Within TELM, these responses align with the stages of Concrete Experience, Disorienting Dilemma, Self-Examination, and early Recognition of Shared Discontent, during which learners face unfamiliar expectations and recognize gaps between prior assumptions and current performance demands (19, 20). Emotional imbalance is widely recognized as a catalyst for perspective transformation, sparking cognitive and emotional shifts that support later growth. For international students, these disruptions were intensified by linguistic and cultural differences, such as unfamiliar terminology, divergent communication norms, and mismatched instructional expectations (2, 10). Significantly, our findings more clearly demonstrate than existing literature that these tensions arise not during clinical placements but within the skills laboratory. This aligns with emerging qualitative evidence suggesting that preclinical learning environments can trigger early transformative processes when learners first encounter unfamiliar norms (29). Therefore, the laboratory serves as the earliest setting where international students begin transformative meaning-making and identity negotiation.

These findings can be further understood through complementary perspectives from international education research. Berry’s acculturative stress framework holds that individuals navigating unfamiliar cultural environments experience psychological strain as they adapt to new norms, values, and expectations (33). In our study, students’ accounts of performance anxiety, uncertainty about classroom behavior, and fear of judgment closely parallel the stress responses described in acculturation literature (34), supporting the interpretation that early laboratory encounters constitute a form of educational acculturation. Additionally, the concept of second-language cognitive load helps explain why international students faced heightened difficulty learning psychomotor skills (35). When learners must simultaneously process procedural instructions, medical terminology, and interpersonal communication in a non-native language, the resulting cognitive burden can impair working memory capacity, reduce attentional resources available for skill execution, and amplify performance anxiety. This dual demand, linguistic processing alongside technical performance, was a recurring pattern in participants’ narratives and may partly account for the intensity of the disorienting experiences reported during early laboratory sessions. By situating these findings within acculturative stress and cognitive load perspectives alongside the experiential–transformative framework of TELM, this study offers a more theoretically layered understanding of the “international” dimension of simulation-based learning.

Instructor feedback and peer support proved powerful tools for reducing early uncertainties and fostering reorganized understanding. In TELM, this process reflects the shift from Self-Examination to Critical Assessment of Assumptions, aligning with Kolb’s Reflective Observation stage (19) and Mezirow’s focus on questioning previous interpretations (20). Recent research highlights the importance of timely, dialogic feedback in fostering reflection and skill development among culturally diverse learners (13, 17, 30). Our findings add to this evidence by showing that reflective transformation happens during preclinical laboratory practice, where feedback helps students reinterpret mistakes, adjust expectations, and begin exploring initial role behaviors. Therefore, within TELM, feedback and peer collaboration are not just supplementary aids but crucial for development, helping learners move from emotional uncertainty to reflective insight and active role participation.

Visual and multimodal materials also played a crucial role in helping students understand psychomotor procedures. In TELM, this reflects the shift from Reflective Observation to Abstract Conceptualization, where learners reorganize fragmented experiences into coherent procedural frameworks (19). It also aligns with Mezirow’s stage of Questioning Assumptions, as students use visual scaffolds to reinterpret expectations and correct misunderstandings caused by linguistic limitations (20). Previous research has shown that multilingual and visually structured materials reduce ambiguity and support understanding among international students (11, 14), with newer studies emphasizing the importance of culturally accessible visual supports for accuracy and fewer miscommunications (5, 31, 32). Our findings build on this by demonstrating that visual supports reduce anxiety, increase psychological safety, and serve as early developmental catalysts within TELM, enabling both cognitive restructuring and emotional stabilization, an interaction rarely emphasized in past research. In this way, multimodal scaffolding functions as a dual-purpose tool that enhances conceptual clarity while also promoting emotional safety during early learning.

As students become more familiar with procedures, they increasingly apply their reconstructed understanding to new situations, experiment with different techniques, and show early signs of adopting a professional role. In TELM, this aligns with the Active Experimentation and Transformative Action stages, where learners begin enacting newly developed understandings and “trying on” emerging professional identities (19, 20). Although previous research usually places professional identity development during clinical placements (8, 9), our findings demonstrate that these shifts occur earlier in the skills laboratory a low-stakes environment that offers repetition, modeling, psychological safety, and opportunities for self-assertion (5, 31, 32) Therefore, the skills laboratory should be viewed not only as a space for technical training but also as a crucial environment for shaping the identity of international nursing students.

Participants also emphasized the combined importance of visual materials, timely feedback, structured instructional guidance, and peer support in facilitating learning. These elements align with TELM’s mapping of the transition from Reflective Observation to Abstract Conceptualization and later Active Experimentation. Consistent with previous evidence, multilingual supports improve understanding (14). Visual scaffolds decrease ambiguity, and guided instruction aids academic adjustment (17, 30). This study extends earlier work by framing these supports as mechanisms that speed up progress through TELM’s developmental phases. Audiovisual scaffolds and structured guidance not only clarified procedural reasoning but also created a psychologically safe space for trial and error, enabling learners to move more confidently into transformative stages. Therefore, instructional supports serve as key drivers of development within TELM rather than merely as pedagogical tools.

Despite these facilitators, students reported that language barriers, cultural differences, performance anxiety, and technological challenges limited early engagement. Within TELM, these align with Initial Confrontation and Self-Examination, where learners experience emotional discomfort and reassess their sense of competence. Prior studies confirm that linguistic and cultural barriers remain persistent challenges for international nursing students (7, 10). Similar findings indicate that language limitations hinder participation (15). Early culture shock disrupts expectations. This study expands this evidence by showing that such disruptions occur earlier than previously documented during initial laboratory encounters. Importantly, students noted that structured support systems helped them see challenges as shared rather than personal shortcomings, facilitating their transition to later transformative stages. Overall, these findings suggest that a well-designed laboratory environment can buffer early linguistic and cultural stressors, turning potential barriers into shared developmental experiences.

However, it is important to consider whether repetitive psychomotor training inherently promotes transformative learning or, under certain conditions, may reinforce instrumental skill acquisition without deeper reflective engagement. Mezirow distinguished between instrumental learning, which focuses on task-oriented problem solving, and emancipatory learning, which involves critical reflection on assumptions and perspective change. In the present study, not all participants demonstrated the same depth or pace of transformation. While the majority of students described shifts in self-perception, growing professional identification, and reinterpretation of early challenges, some narratives remained more focused on procedural mastery and performance improvement without explicit evidence of deeper perspective change. This variation suggests that repetitive practice may be a necessary but not sufficient condition for transformative learning; the presence of structured reflection opportunities, dialogic feedback, and psychologically safe peer interactions appears to distinguish instrumental skill development from early transformative processes. Future studies should explore individual differences in transformative readiness, including the potential roles of prior educational experiences, language proficiency levels, and personal disposition toward reflective engagement, to better understand why some learners move toward transformation while others remain at the level of procedural competence.

Taken together, this study shows that international students’ learning of psychomotor skills is not a simple technical process, but a dynamic journey influenced by emotional upheaval, reflective meaning-making, conceptual restructuring, and the development of professional identity. TELM enhances theoretical understanding by demonstrating that transformative processes begin within the skills laboratory rather than during clinical placements and that early learning settings significantly impact students’ emotional, cognitive, and cultural adaptation. These findings emphasize the need to redesign early laboratory curricula in nursing programs to include intentional language support, culturally responsive teaching, and psychologically safe learning environments. Ultimately, TELM offers a new, evidence-based framework for rethinking early psychomotor training in global nursing education, positioning the skills laboratory as the key place where transformation, identity formation, and procedural mastery initially come together.

Beyond these implications, it is worth considering whether the developmental mechanisms described by TELM are specific to international students or potentially applicable to broader student populations. Several core processes captured in the model, such as initial disorientation, self-examination, reflective reinterpretation, active experimentation, and emerging professional identity formation are likely relevant to novice learners encountering psychomotor skill training for the first time. However, the experiences of international students highlight how cultural linguistic challenges may intensify these processes. Language barriers, unfamiliar pedagogical norms, and cross-cultural adjustment can function as disorienting dilemmas that deepen reflective engagement and meaning making. Therefore, while the overall structure of TELM may have broader applicability, the model is currently grounded in the experiences of culturally and linguistically diverse learners. Future research may examine whether and how the mechanisms described by TELM operate among domestic students or in other educational contexts.

## 5 Conclusion

This study suggests that psychomotor skill development among international nursing students follows a developmental trajectory involving early transformative processes, rather than purely technical skill acquisition, that begins earlier than previously recognized. The findings indicate that the skills laboratory represents the initial educational context in which students encounter unfamiliar expectations, experience disorientation, and begin reconstructing their assumptions about learning and professional practice. This developmental process is conceptualized within the Transformative Experiential Learning Model (TELM).

Structured laboratory experiences incorporating visual scaffolding, timely and constructive feedback, culturally responsive instruction, and peer support were found to facilitate reflective learning, conceptual reorganization, and early professional identity formation. Beyond enhancing procedural competence, these elements promoted psychological safety and emotional regulation, enabling learners to engage more confidently with increasingly complex learning demands.

Although language barriers, cultural differences, performance anxiety, and unfamiliar medical terminology posed significant challenges during early learning, the structured and supportive nature of the laboratory environment mitigated their impact. Students increasingly interpreted these challenges as normative developmental experiences rather than personal inadequacies, highlighting the importance of intentionally designed early simulation-based education.

Several limitations should be acknowledged. First, this study was conducted at a single institution, which may limit the transferability of the findings to other educational contexts. Second, 85% of the participants were from Turkmenistan, resulting in a relatively concentrated cultural distribution. Although this composition reflects the actual demographic profile of international nursing students at the study site and maximum variation sampling was employed across other characteristics (age, gender, academic standing, length of residence), the predominance of one national background may limit the cultural variability captured in participants’ experiences. Readers should therefore exercise caution when generalizing these findings to broader international student populations with different linguistic and cultural profiles. Third, the study relied on self-reported interview data without observational triangulation of actual laboratory performance, which may introduce social desirability or recall bias. Fourth, the cross-sectional design captured a single point in the learning trajectory; longitudinal follow-up would be needed to determine whether the early transformative processes identified here persist or deepen during subsequent clinical placements. Finally, although reflexive strategies were employed throughout, the insider positioning of the research team as faculty members at the same institution may have influenced data collection and interpretation despite deliberate efforts to mitigate this.

Overall, the findings suggest that early transformative processes in nursing education can begin within simulation-based education prior to clinical placement. This underscores the need for educators to reconceptualize early psychomotor training as a critical developmental phase in health professions education. Future research should involve larger and more culturally diverse samples, examine longitudinal learning trajectories across preclinical and clinical contexts, and incorporate educator perspectives to further inform the design of equitable, supportive, and transformation-oriented educational environments.

## Data Availability

The data analyzed in this study is subject to the following licenses/restrictions: Requests to access these datasets should be directed to Hilal Yıldız Çelik, hilal.yildiz@acibadem.edu.tr.
